# Cardio-renal syndrome

**DOI:** 10.12688/f1000research.8004.1

**Published:** 2016-08-31

**Authors:** Joseph Gnanaraj, Jai Radhakrishnan

**Affiliations:** 1 Department of Cardiology, Bridgeport Hospital, Bridgeport, CT, USA; 2 Columbia Presbyterian Medical Center, New York, NY, USA

**Keywords:** Cardiorenal syndrome, heart, kidneys, dysfuntion

## Abstract

Cardio-renal syndrome is a commonly encountered problem in clinical practice. Its pathogenesis is not fully understood. The purpose of this article is to highlight the interaction between the cardiovascular system and the renal system and how their interaction results in the complex syndrome of cardio-renal dysfunction. Additionally, we outline the available therapeutic strategies to manage this complex syndrome.

## Definition

Cardio-renal syndrome (CRS) refers to conditions where acute or chronic dysfunction of either the heart or the kidneys leads to dysfunction of the other. The current definition of CRS encompasses five subtypes that reflect the primary and secondary pathology, the time frame, and simultaneous cardiac and renal co-dysfunction secondary to systemic disease ^1– 3^. These subtypes themselves do not indicate the underlying pathologic mechanism causing the heart and kidney interaction but just represent the primary organ dysfunction that leads to CRS. The type V subtype does not fit under the above definition of CRS, as the kidney and heart dysfunction in this subtype is the result of various systemic illnesses.

The incidence of CRS depends on the subtype. Acute kidney injury (AKI) occurs in 25% to 33% of acute decompensated heart failure (ADHF), which is an independent risk factor for prolonged hospitalization, need for renal replacement therapies, readmission, increased stroke risk, and mortality ^4^. In 60% of ADHF cases, AKI can be seen as an exacerbation of previously diagnosed chronic kidney disease (CKD), whereas in chronic heart failure (HF), CKD has been reported as a comorbidity in 26% to 63% of sufferers ^5^.

## Classification of CRS

The classification of CRS is outlined in Table 1. In this section, we briefly describe each type of CRS, their epidemiology, and their impact on clinical outcomes.

**Table 1.** Types of cardio-renal syndromes.

Type I	Acute heart failure (HF) results in acute kidney injury (AKI) (previously called acute renal failure)
Type II	Chronic cardiac dysfunction (e.g. chronic HF) causes progressive chronic kidney disease (CKD) (previously called chronic renal failure).
Type III	Abrupt and primary worsening of kidney function due, for example, to renal ischemia or glomerulonephritis causing acute cardiac dysfunction, which may be manifested as HF
Type IV	Primary CKD contributes to cardiac dysfunction, which may be manifested as coronary disease, HF, or arrhythmia
Type V (secondary)	Acute or chronic systemic disorders (e.g. sepsis or diabetes mellitus) that cause both cardiac and renal dysfunction

### Type I CRS

Acute impairment of cardiac function leading to renal dysfunction occurs in approximately 25% to 33% of patients admitted with ADHF, depending on the criteria used, with important implications for diagnosis, prognosis, and management ^6^. In ADHF, AKI is associated with increased risk for both short- and long-term all-cause and cardiovascular mortality ^7, 8^. In a cohort of 467 patients admitted with ADHF, patients with persistent renal insufficiency defined as an increase in serum creatinine ≥0.5 mg/dL beyond 30 days had increased mortality (46.1% vs. 20.5%) compared with patients who had a transient rise in creatinine with return to baseline in less than 30 days ^9^.

In acute HF, AKI appears to be more severe in patients with impaired left ventricular (LV) ejection fraction, with an incidence of 70% in patients with cardiogenic shock ^10^. When renal function declines more severely (increase in creatinine of >0.5 mg/dL in combination with >25% increase in serum creatinine level between admission and discharge), 180-day mortality is significantly increased by 10%. Relief of congestion in acute HF with a decrease in N-terminal prohormone of brain natriuretic peptide (NT-proBNP) levels by more than 30% is associated with a 15% absolute lower mortality ^11, 12^.

### Type II CRS

Chronic HF is thought to predispose to CKD. However, chronic HF and CKD commonly coexist, and it is difficult to determine which of the two disease processes is primary ^13^. In the Digitalis Investigation Group trial, pre-existing CKD was found in 45% of chronic HF patients and was associated with a higher rate of hospitalization and death ^14^. In a pooled data analysis from two longitudinal, community-based datasets from the Atherosclerosis Risk in Communities (ARIC) study and the Cardiovascular Health Study, 7.2% of cardiovascular disease (CVD) patients had decline in kidney function when defined as an increase in serum creatinine ≥0.4 mg/dL and 5.6% developed new CKD during an average follow-up period of 9.3 years ^15^.

### Type III CRS

Type III CRS is less well studied, and the prevalence of this syndrome is unknown. It is defined as acute worsening of kidney function that leads to acute cardiac injury and/or dysfunction, such as acute myocardial infarction, congestive heart failure (CHF), or arrhythmia. Cardiac injury may be directly induced by inflammatory mediators, oxidative stress, and upregulation of neuroendocrine systems early after AKI ^16, 17^.

AKI may be associated with volume overload, retention of uremic solutes, pulmonary edema, and cardiac arrhythmias. Acidosis from uremia produces pulmonary vasoconstriction, which can significantly contribute to right-sided HF ^18^.

### Type IV CRS

Primary CKD may contribute to a reduction in cardiac function from cardiac remodeling, LV diastolic dysfunction, hypertrophy, and/or an increased risk for cardiovascular events, such as myocardial infarction, heart failure, or stroke. Independent of age and conventional risk factors, CKD has been shown to be an independent predictor of CVD ^19^. In a study involving 1,120,295 adults, Go *et al*. demonstrated that the adjusted hazard ratio for CVD was 1.4 with an estimated glomerular filtration rate (GFR) of 45–59 mL/min/1.73 m ^2^ (95% confidence interval [CI] 1.1–1.2) compared with 3.4 (95% CI 3.1–3.8) for an estimated GFR of <15 mL/min/1.73 m ^2^ ^20^. In the HEMO study, ischemic heart disease was implicated in 61.5% of cardiac deaths in 1846 chronic hemodialysis patients ^21^. In a systematic review of 13 studies that reported both cardiovascular and all-cause mortality in non-dialysis-dependent CKD patients, increased risk for all-cause mortality was largely driven by cardiovascular deaths (58% of deaths) ^22^.

### Type V CRS

As indicated before, type V CRS is characterized by an acute or chronic systemic illness that concurrently induces cardiac and kidney injury and/or dysfunction. Heart or kidney dysfunction as defined under the umbrella term CRS is not the primary etiology in this subtype. The data are limited on the epidemiology of type V CRS. Common conditions that lead to dysfunction of both kidneys and heart include, but are not limited to, sepsis, drugs such as cocaine, heroin, and chemotherapeutic drugs, infections such as hepatitis B, hepatitis C, and HIV, systemic lupus erythematosus, diabetes mellitus (DM), and amyloidosis. Bilateral renal artery stenosis may manifest as recurrent episodes of flash pulmonary edema.

## Predisposing factors

### Obesity

The cardiometabolic syndrome in the absence of frank DM has been associated with a 3- to 7-fold increased risk of CRS type I in a variety of clinical settings ^23^. Obesity-related glomerulopathy has been long described as a condition of hyperfiltration in obese individuals without DM that ultimately leads to CKD and CRS, particularly type II and type IV ^24^. The adipocytes secrete cytokines such as interleukin (IL)-6 and tumor necrosis factor alpha, which are implicated in the progression of both cardiac and renal disease.

### Anemia and nutritional deficiencies

Anemia, cachexia, and nutritional deficiencies result in elevated tumor necrosis factor alpha and other pro-inflammatory cytokines associated with either HF or CKD and may contribute to further damage and fibrosis of the other organ ^25^.

### Hypertension

Elevated blood pressure, in addition to causing direct cardiac and renal injury, also reflects increased sympathetic neurohumoral activation and is associated with increased incidence of worsening renal failure in patients with decompensated CHF ^26^.

### Diabetes

Diabetes, through many mechanisms, contributes to glomerular dysfunction and damage and ultimate loss of functioning filtration units and further contributes to CKD. Endothelial, mesangial, and podocyte injury in the presence of hypertension and DM results in excess quantities of albumin in Bowman’s space; thus, the proximal tubular cells have an increased reabsorption workload. This phenomenon has been suggested to result in apoptosis of renal tubular cells, further nephron loss, and progression of kidney disease. Indeed, albuminuria and gross proteinuria has been consistently associated with the risk of AKI in a variety of settings ^27^.

## Pathophysiology

**Figure 1.**
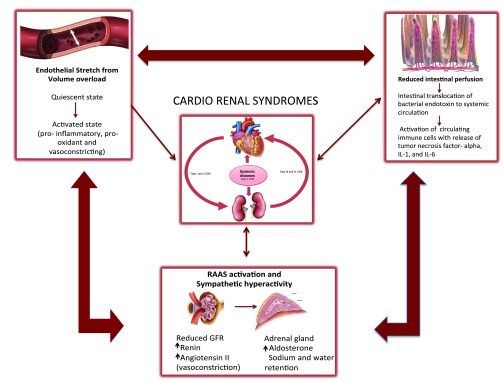
Pathophysiological interactions contributing to cardio-renal syndrome. Figure illustrates how RAAS activation, intestinal hypoperfusion, abnormal endothelial activation, and release of pro-inflammatory cytokines contribute to the development of CRS. CRS, cardio-renal syndrome; RAAS, renin–angiotensin–aldosterone system.

The pathophysiology of CRS is complex and includes dysfunction of the neurohormonal system, abnormal endothelial activation, and release of pro-inflammatory cytokines ( Figure 1). These pathophysiological mechanisms operate simultaneously and sequentially, leading ultimately to cardiac and renal fibrosis and their dysfunction.

### Neurohormonal dysfunction

In type I and type II CRS, venous congestion or reduced cardiac output as a result of cardiac dysfunction reduces GFR. This activates the renin–angiotensin–aldosterone system (RAAS) and nonosmotic release of arginine–vasopressin and other neuroendocrine hormones, such as endothelin, leading to renal injury ^28^. Clinical studies have demonstrated elevated levels of plasma catecholamines in patients with renal dysfunction, indicating sympathetic hyperactivity in type III and type IV CRS ^29^. Afferent signals from diseased kidneys to the central nervous system lead to increased sympathetic nerve discharge and contribute to hypertension, cardiac injury, and further deterioration of renal function. Activation of RAAS increases angiotensin II levels, which promote aldosterone secretion, resulting in sodium and water retention. Angiotensin II also has direct trophic effects on cardiomyocytes and renal tubular cells that promote cellular hypertrophy, apoptosis, and fibrosis ^30^.

### Abnormal endothelial activation

Volume overload from either cardiac or renal dysfunction causes circumferential stretch of endothelial cells. This biomechanical stress activates them, switching their synthetic profile from a quiescent state toward an activated state, which is pro-oxidant, pro-inflammatory, and vasoconstricting ^31^. Concentrations of pro-inflammatory cytokines, such as tumor necrosis factor and IL-6, are increased, which impairs myocardial function and renal function and accelerates HF progression ^32^.

### Endotoxemia, infection, and inflammation

Intestinal hypoperfusion and congestion from cardiac and renal dysfunction leads to intestinal translocation of bacterial endotoxin (lipopolysaccharide [LPS]) into the systemic circulation, which activates circulating immune cells with release of cytokines, such as tumor necrosis factor alpha, IL-1, and IL-6, that can exacerbate myocyte and renal dysfunction ^33^. However, randomized placebo-controlled trials of anti-tumor necrosis factor alpha therapies in patients with CHF (i.e. Randomized Etanercept North American Strategy to Study Antagonism of CytokinEs [RENAISSANCE], Etanercept CytOkine Antagonism in VentriculaR dysfunction [RECOVER], and Anti-Tumor necrosis factor Therapy Against Congestive Heart failure [ATTACH]) have been disappointing and showed no clinical benefit ^34– 36^.

### Role of venous congestion

It is a common notion in the medical community that worsening renal function in HF is due to hypoperfusion. While, hypoperfusion certainly could lead to renal injury, it is not the only mechanism by which HF leads to renal dysfunction. Mullens *et al*. have demonstrated that in patients with low-output decompensated HF venous congestion as suggested by increased central venous pressure (CVP) on admission as well as insufficient reduction of CVP during hospitalization was the strongest hemodynamic determinant for the development of worsening renal function ^2^. There is an inverse relationship between CVP and GFR in CHF ^1^. Elevated CVP leads to elevated renal venous pressure that raises renal interstitial hydrostatic pressure. If interstitial hydrostatic pressure exceeds tubular hydrostatic pressure, tubules will collapse and decrease net ultrafiltration pressure, causing renal dysfunction ^37^.

## Management

Here we describe the general concepts involved in the management of CRS. Often a multidisciplinary, multidimensional systematic and strategic approach is required to prevent and treat CRS.

### Prevention

Since no definitive therapy is available to directly treat any one type of CRS, prevention should be a key strategy. Any patient with dysfunction of either the heart or the kidney is at high risk for the development of CRS. Development of CHF in patients with CVD or risk factors for CVD has an adverse effect on prognosis ^38^. In patients with HF with reduced ejection fraction, the addition of an angiotensin converting enzyme (ACE) inhibitor to conventional therapy has been shown to reduce the incidence of CHF decompensation and HF-related hospitalization ^39^. The addition of RAAS inhibitors in patients with CHF may initially lead to mild worsening of renal function but usually stabilizes and overall is associated with improved mortality ^40^. In patients being treated for decompensated CHF, worsening of renal function may be related to the use of calcium channel blockers and over-diuresis and may not be directly related to the use of RAAS inhibitors ^41^. The use of drugs that directly cause renal injury, such as nonsteroidal anti-inflammatory drugs and contrast agents, should be avoided in patients with or who are at risk for CHF.

Since congestion plays a key role in the pathogenesis of almost all types of CRS, preventing volume overload is a key part of managing patients with CRS. Congestion can set up a vicious cycle of organ dysfunction through multiple pathways, as described under pathogenesis, which in turn leads to worsening cardiac and renal function. Certain symptoms may pre-date the onset of clinical hypervolemia. History of anorexia and early satiety may point toward splanchnic congestion as well as elevated abdominal pressure and ascites, respectively. Prompt identification of these symptoms and attention to volume management will mitigate worsening of cardio-renal dysfunction. History also should focus on the use of nephrotoxic drugs (nonsteroidal anti-inflammatory drugs, antibiotics, high-dose diuretics, etc.). A mean arterial blood pressure of 60 mmHg should be maintained to allow adequate perfusion to vital organs such as the kidney and intestinal tract ^42^. Hypotension, in addition to venous stasis and arteriolar vasoconstriction, can result in intestinal ischemia and release of endotoxins from gut bacteria, activating the immune system and triggering the inflammatory cascade, resulting in cardio-renal dysfunction ^43^.

### Treatment of congestion

Congestion is treated with diuretics and salt restriction. Diuretic dosing is based on renal function and pharmacokinetic properties of diuretics. The DOSE-AHF study demonstrated no significant differences in prognosis or serum creatinine levels between high doses of furosemide (2.5 times their outpatient dose) and lower doses (equal to outpatient dose) in patients hospitalized for ADHF. There was also no benefit of continuous infusion over bolus dosing ^44^.

Once-daily dosing of loop diuretics, such as furosemide, can lead to rebound increase in sodium absorption. Hence, twice-daily dose of loop diuretics should be used. Intravenous loop diuretics should be used in hospitalized patients with ADHF to overcome decreased absorption caused by splanchnic congestion ^45^. When diuretic resistance is encountered, addition of thiazides or mineralocorticoid receptor antagonists (MRAs) can promote diuresis by decreasing the enhanced sodium reabsorption in the distal tubule ^46, 47^. The Cardiorenal Rescue Study in Acute Decompensated Heart Failure (CARRESS-HF) ^48^, a multicenter randomized trial, compared veno-venous ultrafiltration therapy vs. diuretic escalation in patients with either systolic or diastolic HF and worsening renal function (creatinine increase >0.3 mg/dL and evidence of volume overload). When compared with diuretic therapy, ultrafiltration in patients with type I CRS did not show a significant difference in weight loss, mortality, or the rate of hospitalization for HF during the 60-day follow-up period. There was a trend towards increased mortality in the ultrafiltration arm. However, patients requiring inotropes or vasodilator therapy and those with admission creatinine levels >3.1 mg/dL were excluded in this trial. Cardiopulmonary hemodynamics measurements were not made. Hence, it is not known if ultrafiltration has any role in patients who are diuretic resistant. In patients with significant ascites, paracentesis might be useful to reduce intra-abdominal pressure and improve renal hemodynamics and function ^49^.

### RAAS blockade

Excessive salt and water retention from activation of the RAAS in CRS alters cardiac preload and afterload, which further worsens cardiac and renal function. Breaking this cycle could be done by RAAS blockade with an ACE inhibitor and/or angiotensin receptor blocker, preventing further cardio-renal injury. In CHF, RAAS blockade with ACE inhibitors can be given without adverse prognostic significance despite worsening of renal parameters ^50^. Use of beta-blockers may be renoprotective when ACE inhibitors are used for the treatment of CHF ^51^. MRAs such as spironolactone and eplerenone can inhibit neurohormonal surge and prevent worsening of both cardiac and renal function in CRS. However, patients should be carefully monitored for hyperkalemia when given alone or with ACE inhibitors, particularly in the setting of pre-existing renal dysfunction. These drugs can also help overcome loop diuretic resistance when used for hypervolemia ^46^.

### LV assist devices

An LV assist device (LVAD) is an implantable mechanical circulatory support device that has revolutionized the treatment of end-stage HF. Apart from being used to support patients awaiting heart transplant, they are now increasingly being offered to patients ineligible for heart transplant as destination therapy. Hence, LVAD destination therapy can be viewed as an alternative to heart transplant. Pre-LVAD implant renal dysfunction predicts higher mortality after LVAD implantation ^52^. Hence, it is important that patients receive timely referral for LVAD therapy before HF worsens and leads to CKD. Renal function usually improves after LVAD implantation if decreased GFR is due to renal hypoperfusion before implantation. Most of the recovery tends to occur in the first month after LVAD placement and no further improvement in renal function occurs from about 1 month after pump placement. Improvement in renal function after LVAD implantation may be through improvement in intrarenal hemodynamics ^53^ and reversal of renal hypoperfusion.

### What the future holds

Implantable devices to measure intravascular volume in real time are being developed. The COMPASS-HF ^54^ trial showed that increases in intracardiac pressures often arose independently of weight changes, such that monitoring of weight alone was inadequate to identify congestion in time to avert the events associated with HF. Early identification of elevations in pulmonary artery pressures that occur several days to weeks before the onset of worsening signs, symptoms, and hospital admission ^55^ is now possible through implantable pulmonary pressure monitoring systems. This provides an opportunity to provide early intervention by targeting these pressures, which might reduce the risk of CHF decompensation and reduce the incidence of CRS.

Aldosterone plays a major role in cardiac, vascular, and renal remodeling by promoting oxidative stress, inflammation, fibrosis, and hypertrophy ^56, 57^. MRAs may reduce the negative effects of MR activation on the kidney, augment diuresis in diuretic-resistant patients, and attenuate the pre-renal state related to neurohumoral activation in ADHF. Further repeated renal injury by MR activation may contribute to the later progression to CKD. Clinical trials are underway using finerenone, a novel nonsteroidal MRA, in CRS ^58^.

Another agent that has shown promise in CRS is serelaxin, which is a recombinant form of human relaxin-2. Relaxin, a peptide hormone secreted during pregnancy, is associated with increase in cardiac output and decrease in systemic vascular resistance. It was hypothesized that this pharmacological profile might have beneficial effects in ADHF and renal function and was tested in the RELAX AHF trial ^59^. Serelaxin was associated with significantly lower serum creatinine and plasma cystatin C levels in the first 5 days after enrollment. All-cause (7.3% vs. 11.3%, P=0.02) and CV death (6% vs. 9.5%, p=0.028) were lower at 180 days in the serelaxin arm. On subgroup analysis, mortality benefits were higher in patients not treated with beta-blockers, with GFR <50 mL/min, and aged >75 years. Adverse events related to renal impairment (6% vs. 9%, P=0.03) were similar between the two arms, with mortality benefit in the serelaxin arm. These results are promising and need to be confirmed in an ongoing large-scale study, the results of which are expected in early 2017.

Recent research has focused on enhancing the effects of natriuretic peptides (NPs) such as atrial NP and BNPs. These NPs contribute to the regulation of sodium and water balance, blood volume, arterial pressure, and sympathetic inhibition through their effects on the venous system, kidneys, and brain. Neprilysin is an enzyme that degrades these NPs. Inhibition of neprilysin increases the physiological effects of NPs. The combined inhibition of both the angiotensin II receptor and neprilysin with a novel drug, LCZ696 (sacubitril/valsartan), was more effective in reducing the risk of death from cardiovascular causes or hospitalization for HF than was ACE inhibition with enalapril in the PARADIGM-HF trial. This favorable response in mortality occurred in the absence of worsening of renal function or hyperkalemia in the LCZ696 group as compared to the enalapril group ^60^. Of note, more than 3000 patients in the study were in stage III CKD with estimated GFR between 30 and 60 mL/min/1.73 m ^2^. Further studies are needed to address the effect of LCZ696 on CRS.

Sympathetic system overactivity plays a key part in the progression of CRS. Catheter-based renal denervation is a promising new therapy designed to reduce renal sympathetic activity, leading to a generalized reduction in systemic sympathetic activation. Based on animal studies, pilot trials are underway to study the effect of renal denervation on the heart and kidney in CHF ^61^.

## Conclusion

CRS is now a firmly established entity relating to the complex interaction that exists between the heart and the kidney. The prevention and treatment of CRS remains a challenge to physicians. Primary prevention strategies focusing on the modification of risk factors such as obesity, hypertension, DM, and tobacco use are important. From a treatment standpoint, hemodynamic support in low-output states, relief of congestion with judicious use of diuretics, and suppression of neurohumoral activation remain the cornerstones of treating CRS. Novel devices and therapeutics such as intracardiac pressure monitoring, neprilysin inhibition, third-generation MRAs, and sympathetic denervation are being investigated.

## Abbreviations

ACE, angiotensin converting enzyme; ADHF, acute decompensated heart failure; AKI, acute kidney injury; CHF, congestive heart failure; CI, confidence interval; CKD, chronic kidney disease; CRS, cardio-renal syndrome; CVD, cardiovascular disease; CVP, central venous pressure; DM, diabetes mellitus; GFR, glomerular filtration rate; HF, heart failure; IL, interleukin; LVAD, LV assist device; NT-proBNP, N-terminal prohormone of brain natriuretic peptide; RAAS, renin–angiotensin–aldosterone system.
